# Re‐parameterization of a mathematical model of African horse sickness virus using data from a systematic literature search

**DOI:** 10.1111/tbed.14420

**Published:** 2022-01-12

**Authors:** Emma L. Fairbanks, Marnie L. Brennan, Peter P. C. Mertens, Michael J. Tildesley, Janet M. Daly

**Affiliations:** ^1^ School of Veterinary Medicine and Science University of Nottingham Nottingham UK; ^2^ The Zeeman Institute for Systems Biology & Infectious Disease Epidemiology Research School of Life Sciences and Mathematics Institute University of Warwick Coventry UK

**Keywords:** African horse sickness, mathematical model, vector‐borne disease

## Abstract

African horse sickness (AHS) is a vector‐borne disease transmitted by *Culicoides* spp., endemic to sub‐Saharan Africa. There have been many examples of historic and recent outbreaks in the Middle East, Asia and Europe. However, not much is known about infection dynamics and outbreak potential in these naive populations. In order to better inform a previously published ordinary differential equation model, we performed a systematic literature search to identify studies documenting experimental infection of naive (control) equids in vaccination trials. Data on the time until the onset of viraemia, clinical signs and death after experimental infection of a naive equid and duration of viraemia were extracted. The time to viraemia was 4.6 days and the time to clinical signs was 4.9 days, longer than the previously estimated latent period of 3.7 days. The infectious periods of animals that died/were euthanized or survived were found to be 3.9 and 8.7 days, whereas previous estimations were 4.4 and 6 days, respectively. The case fatality was also found to be higher than previous estimations. The updated parameter values (along with other more recently published estimates from literature) resulted in an increase in the number of host deaths, decrease in the duration of the outbreak and greater prevalence in vectors.

## 1 INTRODUCTION

African horse sickness (AHS) is caused by African horse sickness virus (AHSV) of the genus *Orbivirus* in the family *Reoviridae*. Endemic to sub‐Saharan Africa, it often emerges when periods of heavy rain follow hot and dry conditions. This favours its principle vector, *Culicoides* spp., with *Culicoides imicola* usually considered to be the most important vector species in Africa (Mellor & Hamblin, 2004). AHSV hosts include horses, mules, donkeys and zebras. Zebras, the only equids native to South Africa, are believed to be the reservoir host (Barnard, 1998).

There are nine immunologically distinct serotypes of AHSV defined on the basis of antigenic reactivity of antibodies to the outer capsid virus protein VP2 (Bachanek‐Bankowska et al., 2014). In endemic regions, usually only one serotype circulates at a time. Historically, serotype 9 was responsible for epizootics of AHS outside Africa. Outbreaks in central and East Africa have occasionally spread to Egypt, the Middle East and southern Arabia (Mirchamsy & Hazrati, 1973). A major epidemic in 1959−1961, spread through the Near East and Arabia as far as Pakistan and India, resulted in the death of an estimated 300,000 equids (Anwar & Qureshi, 1973; Howell, 1960). A further epidemic of AHSV in northwest Africa (Morocco, Algeria and Tunisia) in 1965−1966 spread briefly to southern Spain but was eliminated by vaccination and by killing infected equids (Hazrati, 1967). The first occurrence of an outbreak outside Africa not caused by serotype 9 was in July 1987, when serotype 4 was reported in central Spain (Lubroth, 1988). The virus overwintered and caused further outbreaks in southern Spain, Portugal and Morocco in subsequent years before it was eliminated in 1991 (Baylis et al., 1997; Portas et al., 1999; Rodriguez et al., 1992). In 2007, outbreaks of AHS in West Africa were caused by serotype 2 (Nigeria and Senegal) and serotype 7 (Senegal) (Diouf et al., 2013). Outbreaks of AHS have recently been reported in Ethiopia (Aklilu et al., 2014), Thailand (Lu et al., 2020) and Malaysia (Castillo‐Olivares, 2021).

AHS presents as acute, subacute or subclinical forms (Carpenter et al., 2017). In naive populations of horses, case fatality may exceed 90% in epidemics (Castillo‐Olivares, 2021). The acute respiratory form is characterized by a short incubation period (3−5 days), and the animal usually dies from severe hypoxia, congestive heart failure or a combination of both after around 1 week. The subacute or cardiac form of AHS has an incubation period of 1−2 weeks with a short fever followed by the classical clinical sign of AHS and oedema of the supraorbital fossae. The case fatality is around 50% with death usually occurring within 1 week. In most outbreaks, a mixed pulmonary and cardiac form is most commonly seen, which causes fatality of around 80% of susceptible equids (Theiler, 1921). The subclinical form, often referred to as African horse sickness fever, is common in zebras, African donkeys and horses that are partially immune because they have been vaccinated or have recovered from a previous infection (Lu et al., 2020). The outbreaks of AHSV in Spain in 1987 and Thailand in 2020 were associated with importation of (infected) zebras from Africa (Grewar et al., 2021; Rodriguez et al., 1992).

In order to model the risk posed by AHSV if it emerges in countries where equids have no prior exposure, we need data on various parameters such as the length of the latent and infectious periods. It is also important to know whether there is any association between the serotype causing an outbreak and the values of these parameters. In the case of AHSV, parameters such as the latent period are not possible to determine in the field as the exact time of infection is generally not known. Here, a systematic search and data extraction, focusing on studies documenting experimental infection of naive equids in vaccination trials, was performed to inform a model for AHSV previously suggested by Backer and Nodelijk (2011). Overall, 26 studies were used to derive parameters describing the host–virus interactions, compared to the three studies used in the development of the Backer and Nodelijk model (J. A. House et al., 1994; Roy et al., 1996; Scanlen et al., 2002). Parameters derived from the review were the time until the onset of viraemia, clinical signs and death after experimental infection of a naive equid as well as the duration of viraemia. It is important to consider the role of vectors when modelling AHSV. The parameters for the vectors were updated from literature, similarly to Gubbins et al. (2014); some of this literature was published after the Backer and Nodelijk model.

AHSV is listed as a notifiable disease in disease‐free countries by the World Organisation for Animal Health (OIE) (2021). An outbreak can have a severe impact on the welfare of equids and be disruptive for the equine industry (Allison et al., 2009; Clemmons et al., 2021). Policy makers with responsibility for deciding the course of action if an outbreak occurs in a previously disease‐free country are heavily reliant on models to predict the likely outcome of outbreaks (Daly et al., 2013; Grassly & Fraser, 2008).

## 2 METHODS

### 2.1 Systematic literature search

A systematic search (limited to the title and abstract) was performed (by E. L. Fairbanks and J. M. Daly) using PubMed with the search terms ‘African horse sickness virus’ AND ∼vaccine AND (∼challenge OR ∼trial)’ and ‘African horse sickness virus’ AND ‘experimental infection’, where ∼ means terms including and similar too. Eligible studies included the inoculation of a naive equid with AHSV. The citations and references of eligible articles found on PubMed were then searched in order to find further relevant articles. A similar search term was used to search CAB abstracts; however, no additional research articles were identified. Supporting Information 1 describes how vector‐related parameters were updated, also by systematic searches.

#### 2.1.1 Data extraction and analysis

Studies were eligible for data extraction if they gave a value for the time until onset of viraemia and/or clinical signs and/or death. To determine whether these attributes were significantly different between the virus serotypes, a Kruskal–Wallis test was used. This is a non‐parametric method for testing whether samples originate from the same distribution, that can be used for comparing more than two independent samples. This analysis was performed using the stats R package version 4.2.0 (R Core Team and contributors worldwide, 2021). Data for the unidentified serotypes were not included in this analysis. Also, analysis for the time until viraemia was repeated including only serotypes with more than one data value. We also performed this statistical analysis to determine whether infection method (either intravenous or subcutaneous inoculation) had an impact on these disease characteristics. A paired and two‐sample t‐tests were used to determine if there was a difference between the estimated onset of viraemia between polymerase chain reaction (PCR)‐based and virus isolation methods in the equids for which both were compared and all equids for which data for the onset of viraemia were analyzed (including both PCR and virus isolation for equines with both available), respectively.

### 2.2 Re‐parameterization of a mathematical model

**FIGURE 1:**
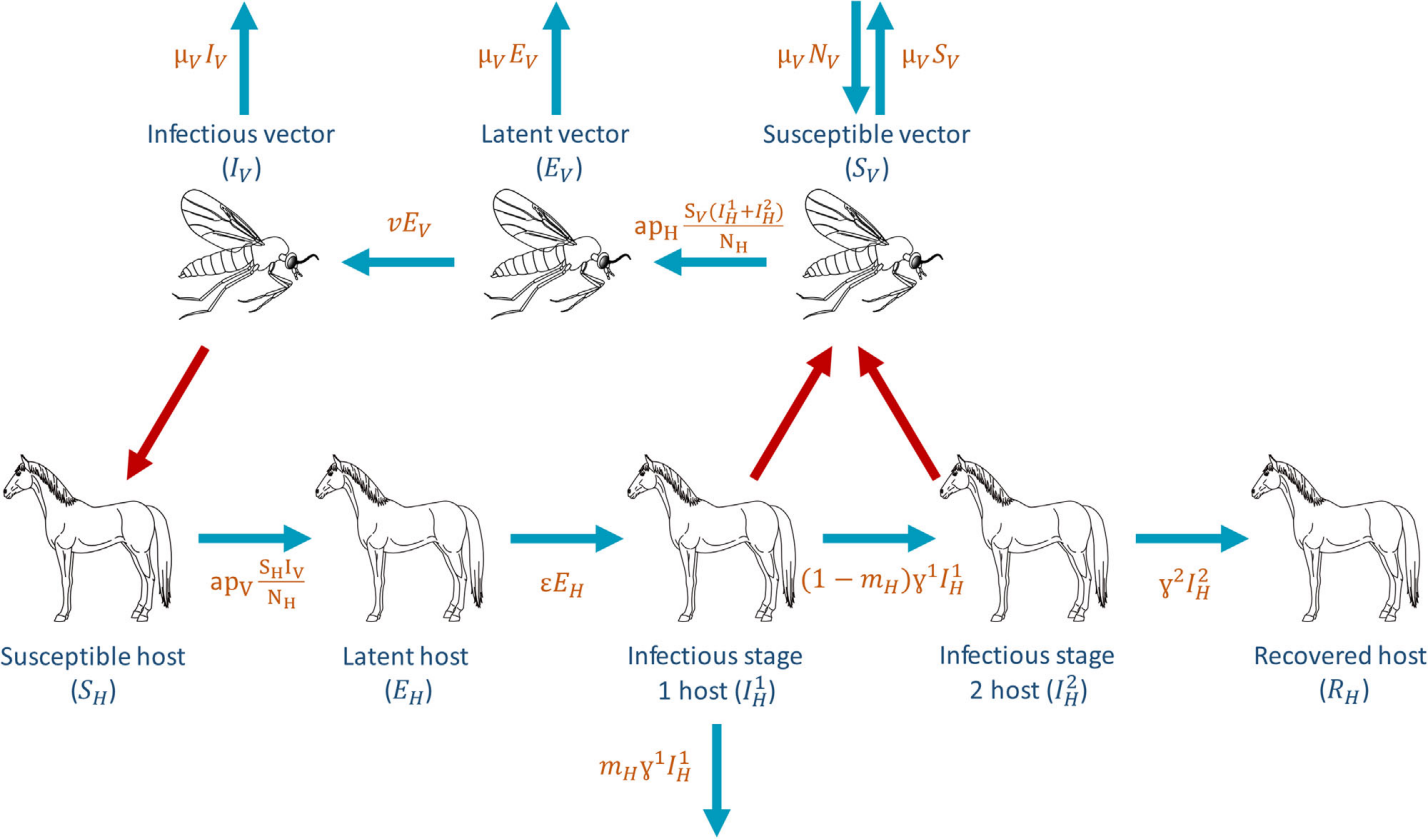
The infection cycle of African horse sickness virus (AHSV). Blue arrows represent transfers between compartments with their rates given in orange. Red arrows represent between species transmission

We will consider a deterministic ordinary differential equation (ODE) model for AHSV suggested by Backer and Nodelijk (2011). In the Backer and Nodelijk model, the vector population adapts to changes in the vector:host ratio. Here, we do not include this in the model. This is due to the assumption that the size of the vector population is dependent on the carrying capacity of midges in the environment surrounding the horses rather than the number of horses present. The total number of vectors therefore does not change. We assume the number of hosts present is likely to reflect the size of the premises. Figure 1 shows the infection diagram for this system. Details of the model along with the ODEs are given in Supporting Information 2.

Parameters updated included the latent period, infectious period of both dying and surviving equids and host fatality. The infectious period of surviving hosts was calculated using data from live virus isolation, similarly to Backer and Nodelijk (2011). Here, we set a conservative estimate of the case fatality of 0.84; however, sensitivity analysis allows us to observe the effect of this value on model outputs (Supporting Information 3). The latent period and infectious period of dying and surviving hosts are divided into multiple stages; this allows them to have a gamma distribution. For example, the total duration of the latent period follows a gamma distribution with mean duration *ε* and variance *ε^2^/i*, where *i* is the number of stages.

We compare simulation results for this model for the parameters published in Backer and Nodelijk (2011) and the updated parameters. ODEs were integrated using the MATLAB function *ode45*. Sensitivity analysis was performed on the model with an updated range of possible parameter values. This was done on the duration of the outbreak, total number of infected equine hosts during the outbreak and the basic reproduction number (*R*_0_) using the partial rank correlation coefficient (PRCC) method (Supporting Information 3).

## 3 RESULTS

### 3.1 Systematic literature search

**FIGURE 2:**
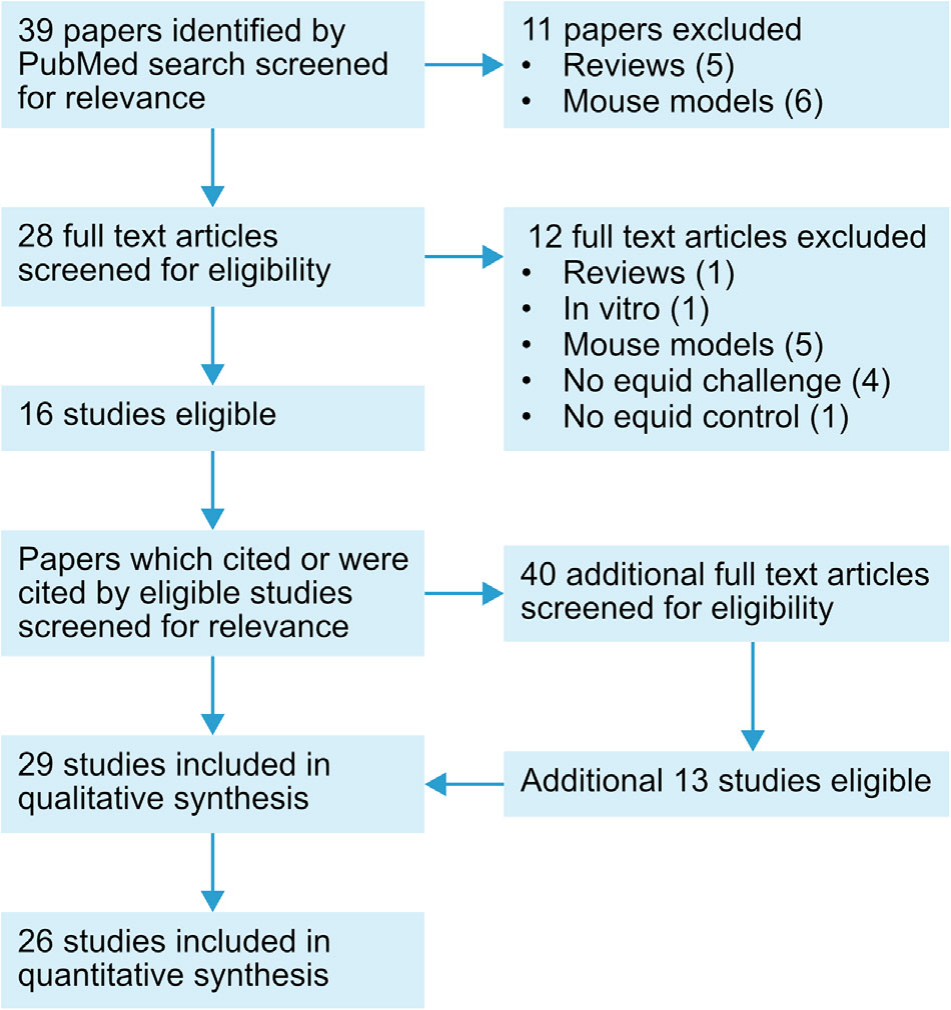
PRISMA flow diagram describing the process of the systematic search

A total of 39 articles were found during the PubMed search performed on 20 May 2020. Of these, 11 were excluded during a first screening of their titles for being narrative reviews or a non‐equid experimental infection. After reading the full texts of the remaining 28 articles, a further 12 were excluded for being narrative reviews, in vitro experiments, non‐equid infection experiments, not challenging equids or not using unvaccinated control animals, all of which mean that no naive equids were experimentally infected. The citations and references of the eligible 16 articles found on PubMed were then screened in order to find more relevant articles. A total of 40 additional full‐text articles were then read and 13 were found to be eligible under the same inclusion criteria. During the screening, six titles were identified that qualified for a full text read but could not be accessed; these were not included in the qualitative synthesis. The process of the literature search is described by the PRISMA flow diagram in Figure 2 (Liberati et al., 2009).

**FIGURE 3:**
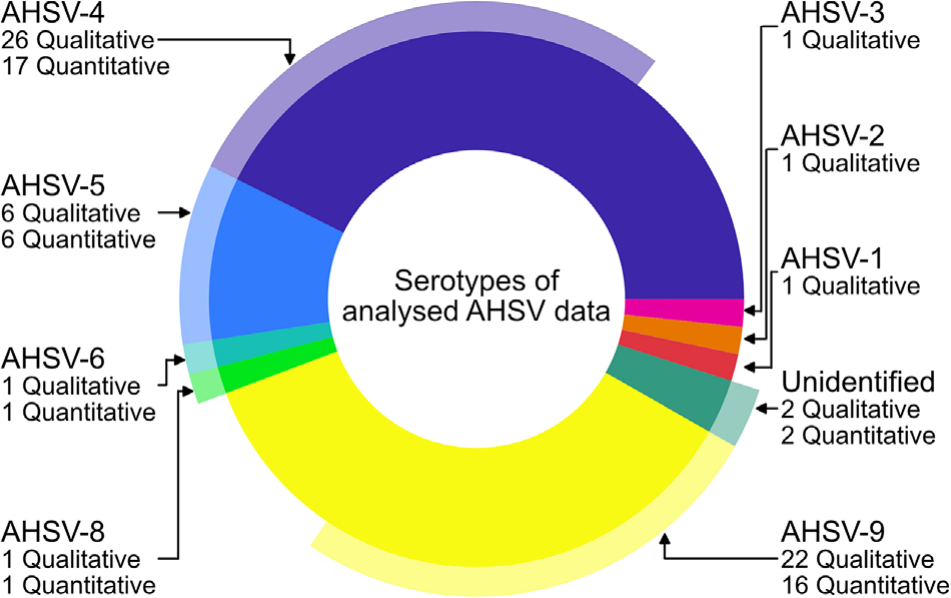
Pie chart showing the percentage of qualitative samples of each serotype from the systematic search (inner circle) and the percentage of these used in the quantitative analysis after data extraction (outer circle)

Altogether, 29 studies were found recording the experimental infection of 61 naive equids; 53 horses, five donkeys and three mules (Alberca et al., 2014; Alexander & Du Toit, 1934; Du Plessis et al., 1998; Dubourget et al., 1992; El Hasnaoui et al., 1998; Guthrie et al., 2009; Hassanain, 1992; Hazrati & Ozawa, 1965; C. House et al., 1990; J. A. House et al., 1994, 1992; Lelli et al., 2013; Lulla et al., 2017; Martínez‐Torrecuadrada et al., 1996, 1997; Minke et al., 2012; Mirchamsy & Taslimi, 1964a, 1964b, 1968; Ozawa & Bahrami, 1966; Ozawa et al., 1970, 1965; Quan et al., 2010; Roy et al., 1996; Scanlen et al., 2002; Stone‐Marschat et al., 1996; van Rijn et al., 2018; von Teichman et al., 2010; Whitworth, 1930). These included all serotypes apart from AHSV‐7 and two studies where the serotype was unidentified (Figure 3). The article by Sánchez‐Matamoros et al. (2016) was considered, but not included due to uncertainty of the vaccination status of the 14 experimentally infected horses. Qualitative synthesis of the information available from papers can be found in Table S5. El Hasnaoui et al. (1998) was included in the qualitative analysis due to the naive experimental infections; however, the full text was not accessible so details about values of interest were not available.

#### 3.1.1 Data extraction and analysis

Of the 29 studies analyzed during the qualitative synthesis, 26 were eligible for data extraction and analysis (i.e. gave a value for the time until onset of viraemia and/or clinical signs and/or death).

**FIGURE 4:**
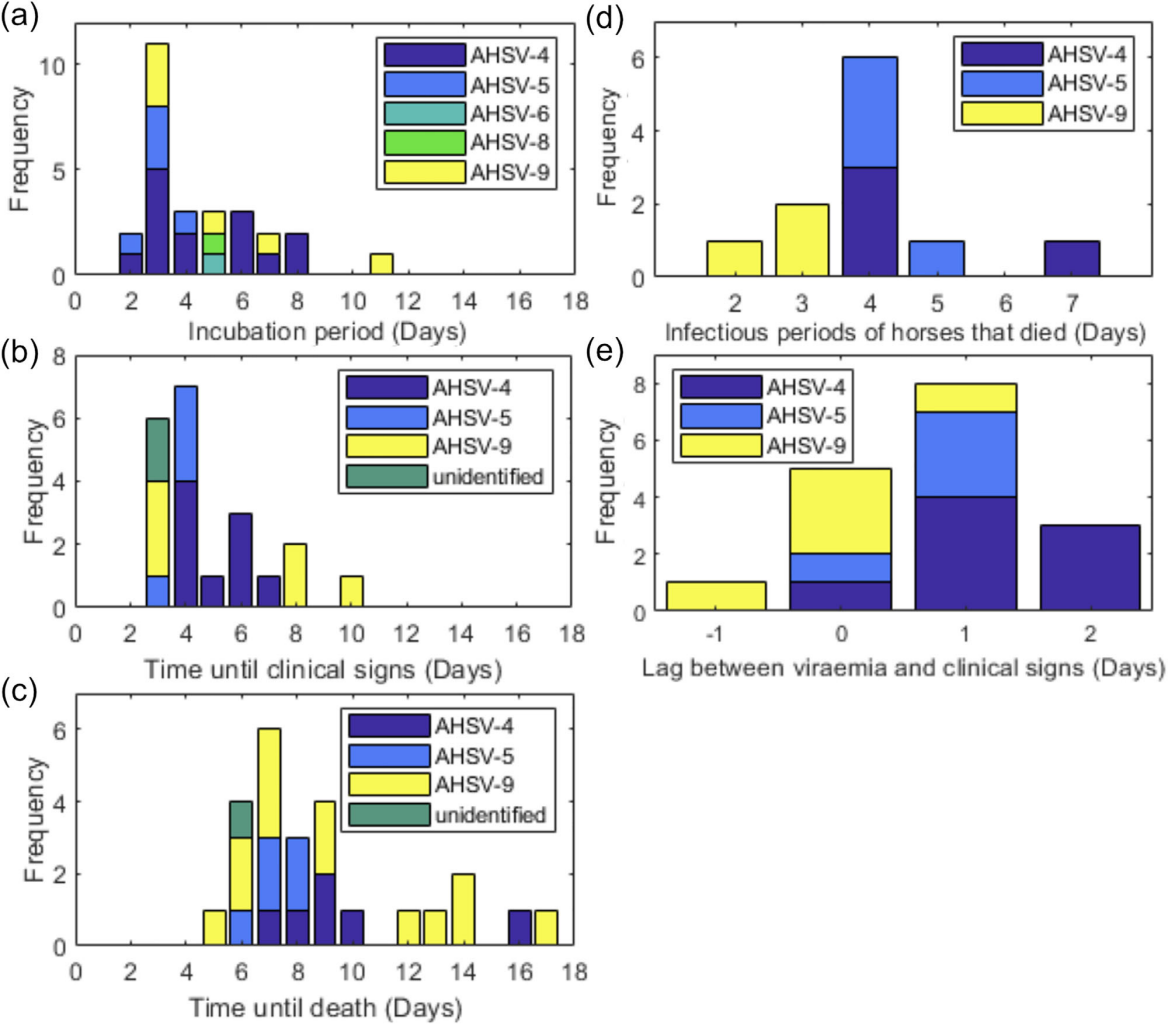
Histograms of (a) the time until viraemia, (b) time until clinical signs, (c) time until death, (d) total infectious period of horses that died and (e) time lag between viraemia and clinical signs for equids experimentally infected with African horse sickness virus (AHSV)

A total of 27 naive experimental infections where the time until viraemia had been measured were analyzed (Figure 4a). These varied from 2 days to 11 days with mean 4.6 days and included serotypes AHSV‐4 (*m* = 14), AHSV‐5 (*m* = 5), AHSV‐6 (*m* = 1), AHSV‐8 (*m* = 1) and AHSV‐9 (*m* = 6). The time until the onset of clinical signs was recorded for 21 of the naive experimental infections for serotypes AHSV‐4 (*m* = 9), AHSV‐5 (*m* = 4), AHSV‐9 (*m* = 6) and two unidentified serotypes. The mean was 4.9 days and individuals varied from 3 days to 10 days (Figure 4b). The mean time until death after experimental infection was 9.1 days and varied between 5 and 17 days (Figure 4c). This was recorded for 25 horses for serotypes AHSV‐4 (*m* = 6), AHSV‐5 (*m* = 5), AHSV9 (*m* = 13) and an unidentified serotype (*m* = 1).

Results from the Kruskal–Wallis tests to determine whether the time to viraemia, onset of clinical signs or death are significantly different between the virus serotypes or inoculation methods are given in Table S6. None of these tests gave a significant result (*p* < .05); therefore, we conclude that there is no evidence that the time until viraemia, the onset of clinical signs or death varies between serotypes or inoculation method. A paired t‐test showed that there was no significant difference between the start of viraemia between PCR and virus isolation methods in the six equids for which both were compared (*t* = −1.55, *df* = 6, *p* = .17). A two‐sample t‐test performed for the 27 equines for which data for the start of viraemia was analyzed also showed no statistically significant difference between detection of genetic material by PCR‐based methods and detection of infectious virus (*t* = 0.64, *df* = 31, *p *= .52).

For 11 horses, the time until viraemia and death due to AHSV was recorded. The total infectious period (difference between onset of viraemia and death) of these horses varied between 2 and 7 days with a mean of 3.9 days (Figure 4d). For three horses that survived experimental infection, the time between the start and end of viraemia was recorded using virus isolation. The total infectious periods of these horses were 4, 5 and 5 days, giving a mean of 4.7 days.

The lag between the start of viraemia and onset of clinical signs was reported for 18 horses. This varied between −1 (horse showed clinical signs before viraemia) and 3 days, with a mean of 0.8 days (Figure 4e).

Whether the equid died of AHSV, was euthanized for ethical reasons or survived was reported for one donkey and 44 horses. The donkey survived the AHSV‐9 experimental infection. Of the 44 horses 31 died, seven were euthanized and six survived. A further eight horses either died naturally due to infection or were euthanized, but this was not specified. If we assumed that the euthanized horses were determined to be ill enough that they would not survive then a total of six horses out of 52 survived and the case fatality is calculated to be 0.88. However, if we do not make this assumption and do not consider the seven horses that were euthanized, six horses out of 37 survived giving a case fatality of 0.84.

### 3.2 Re‐parameterization of a mathematical model

The model parameters updated from the review are given in Table 1. As well as these host parameters, some vector parameters were also updated from the literature. These were the blood feeding interval (frequency of biting) (Mullens et al., 2004), extrinsic incubation period (latent period of midges) (Carpenter et al., 2011), vector life‐span (Gerry & Mullens, 2000) and transmission probability from a host to a vector during a bite (Carpenter et al., 2011). Details of these and other unchanged parameters are given in Supporting Information 1.

**TABLE 1:** Updated model parameters. Time periods are given in days. The parameter values used in Backer and Nodelijk (2011) are described in the previous value column and the parameter values used in our model are described in the updated value column

		Previous value		Updated value	
Parameter	Symbol	Default value	5%−95% range	Default value	5%−95% range
Latent period	1/*ε*	3.7	2.5−4.9	4.6	2−8.5
Number of stages	1	16		5	
Infectious period (dying hosts)	*T*_*inf*_^1^	4.4	2.2−6.6	3.9	2.1−6.9
Number of stages	*n* ^1^	19		11	
Infectious period (recovering hosts)	*T_inf_* ^2^	6.0	3.0−9.0	4.7	4−5
Number of stages	*n* ^2^	10		13	
Host fatality	*m_H_*	0.7	0.43−0.97	0.84−0.88	

We fit a gamma distribution to the quantitative data for the time until viraemia and duration of viraemia to calculate the number of stages in the latent and infectious periods, respectively. The number of stages is equal to the gamma parameter. The number of stages for the latent period and infectious period for dying hosts are 5 (95% CI:3–9) and 11 (95% CI:5–26), respectively. Due to there not being many data points for the infectious period of surviving hosts, the number of stages needed was calculated as 93 (95% CI:19–659). This large value and confidence interval is due to the limited number of data points available. Therefore, due to the limited number of samples, the number of stages for the infectious period of surviving hosts is scaled from the infectious period of the dying hosts. As the mean infectious period of surviving hosts is 1.2 times longer than that of dying hosts, the infectious period of surviving hosts is divided into 13 stages (2 more than the number of stages for the dying horses).

**FIGURE 5:**
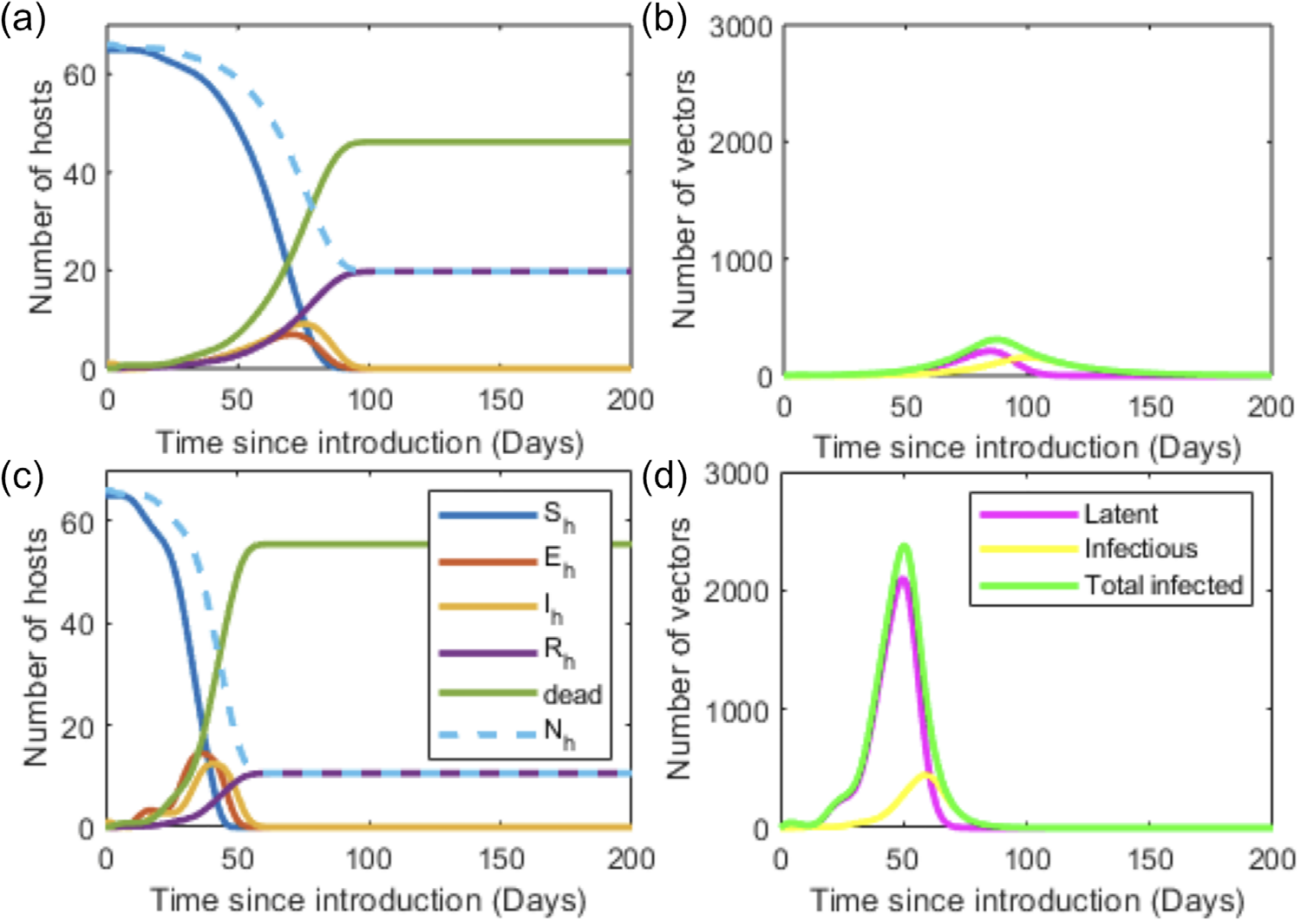
Simulations of the Backer and Nodelijk (2011) model using the published (a, b) and updated (c, d) parameter values showing the outcomes for the host (a, c) and vector (b, d) populations over time after the introduction of infection into a population of 66 horses. These parameter values are given in Table 1 and Table S1

Figure 5 compares the output of the updated parameters and the original Backer and Nodelijk model parameters detailed in Table 1 and Table S1. Initially, one horse is assumed to be in the first infectious stage (*T_inf_* ^1^). The updated parameters suggest the outbreak would have a shorter duration, and the total number of host deaths increased from 46 to 55. For both sets of parameters, all hosts on the premises become infected. The updated parameter values suggest a peak of 13 infectious hosts on day 41 of the outbreak. However, the original parameter values suggest this peak is on day 75 with nine infectious hosts. The peak in the number of infectious vectors comes after this. The updated parameters suggest that the peak is 439 infectious vectors on day 58 of the outbreak compared to 156 infectious vectors on day 100 using the original parameters.

Sensitivity analysis (Supporting Information 3) showed the parameter that most significantly influences the duration of the outbreak is the vector:host ratio, with a larger vector population being associated with shorter outbreaks. Here, the host infectious period (*T_inf_*), vector life‐span (1*/*μ*_V_*), host to vector (*p_H_*) and vector to host (*p_V_*) transmission rates, and the vector:host ratio are associated with outbreaks that spread more rapidly upon emergence. It is also shown that longer host latent periods (1*/ε*), duration between vector bites (1*/a*) and extrinsic incubation periods (1*/ν*) increase the duration of the outbreak. The host case fatality (*m_H_*) did not significantly influence *R*_0_.

## 4 DISCUSSION

In order to better inform the host parameters used in the ODE model developed by Backer and Nodelijk (2011), data were extracted from 26 studies, representing 43 experimentally infected naive equids. Since this analysis, further naive experimental infection studies for AHSV have been published (van Rijn et al., 2020). These data did not conflict with the findings of this study. Overall, the updated model parameters suggest more hosts on the premises would die and the outbreak on each premises would be shorter than previously predicted. More vectors would also become infected, which may increase the probability of transmission between premises.

Many of the studies in this systematic search use different volumes and titres of virus for experimental infection. Experimental infection through needle injection is not a natural route of infection. How well this mimics natural infection by midges is not known (Coetzee et al., 2014; Darpel et al., 2012). It is therefore unclear whether this route of infection or the titre/volume of virus inoculated affects the time taken to become viraemic or show clinical signs. Bluetongue virus experimental infections have shown that infection via the intradermal route in sheep reduced the time until clinical signs and increased their severity compared to the intravenous route (Umeshappa et al., 2011). It was also found that subcutaneous inoculation appears to mimic the natural route of infection more closely than the intravenous inoculation route, in respect to the dissemination of the virus from the skin to secondary target organs as has been observed following natural infection (Coetzee et al., 2014; Umeshappa et al., 2011). In this review, we found no difference in the time until viraemia, clinical signs or death between intravenous and subcutaneous routes of infection.

Variation in VP2 is a mechanism for escape from pre‐existing neutralizing antibodies that block viral attachment to the host cell (Burrage et al., 1993; Jewell & Mecham, 1994). These variations may impact the infectivity of the virus for the cells of hosts or vectors, but viral pathogenicity is typically determined by more than one viral protein and is therefore not necessarily serotype dependent. For example, van Rijn et al. (2018) reported that introducing a deletion in the NS3/NS3a protein of AHSV renders the virus avirulent. Lulla et al. (2016) reported that an AHSV‐1 isolate was more virulent than an AHSV‐4 isolate in mice, but whether this difference was determined by VP2 or indeed consistent between different isolates of the respective serotypes was not addressed by that study. No differences in time until viraemia, clinical signs or death were found between serotypes in this study. Studies of bluetongue virus (BTV) (the type species of the *Reoviridae* family) indicate that more recently identified serotypes (BTV‐25−27) show adaptations involving VP2 (as well as VP1, VP3 and VP7) that support direct‐contact transmission, rather than by vector insects. These changes also influence the virulence of these serotypes for different ruminant hosts (Guimerà Busquets et al., 2021; Pullinger et al., 2016), but there is no evidence for similar variations in AHSV.

Articles analyzed to parameterize this model dated back as far as 1930, that is, before PCR‐based techniques were available. In one study, the reverse transcription‐PCR (RT‐PCR) assay and virus isolation methods were found to be equally sensitive for detection of virus in blood samples from horses experimentally infected with AHSV‐4. However, viraemia was detected more consistently by RT‐PCR than by virus isolation from horses infected with AHSV‐9 except from one animal for which virus was detected only by virus isolation (Sailleau et al., 1997). Other studies have shown that RT‐quantitative PCR has higher sensitivity than virus isolation (Guthrie et al., 2013; Quan et al., 2010). Three articles, with a total of six experimental infections of naive equids from which data were extracted in this study, compared the detection of viraemia by PCR and virus isolation methods. Of these, two studies (involving a total of four equids) (Alberca et al., 2014; Guthrie et al., 2009) did not find a difference in the first day of detection of viraemia, whereas one found that viraemia was detected one day earlier by PCR than by virus isolation in both animals involved (Lelli et al., 2013). Further analysis showed no significant differences between virus detection methods and estimated onset of viraema. However, caution is needed when interpreting RT‐PCR results in relation to the duration of viraemia. For example, in BTV infection, nucleic acid can be detected in blood of hosts after infectious virus has been cleared (MacLachlan, 1994; MacLachlan et al., 1994; Mayo et al., 2021). Consequently, a long duration of viraemia detected by PCR is not a reliable guide to infectivity, or the ability of the host to act as a source of virus to infect feeding insects. The surviving host will have developed neutralizing antibodies that effectively inhibit detection by virus isolation, even if the virus was still viable (which is uncertain).

Many vector‐borne disease models consider daily differences in temperature and seasonality in the vector population. Here, we do not consider seasonality; we simulate the model for the time of year at which midge‐borne diseases are thought to be most likely to emerge in the United Kingdom. The midge species and climates in laboratory/field experiments used to derive the midge parameters may not be an accurate representation of the most likely vector in all regions where there is a risk of AHSV. The use of the Gerry and Mullens (2000) value for midge mortality compared to the Wittmann et al. (2002) value reduces estimates for transmission. A limitation of Wittmann et al. (2002) is that *Culicoides* were examined in the laboratory which may affect their mortality in comparison to wild *Culicoides*. However, the parameter estimated from Gerry and Mullens (2000), based on trap data, also has its limitations. A recent study found that biting rate per day (per cow) was expected to be approximately 50% of a 24‐h trap collection (Möhlmann et al., 2021). Another study found that on average 2.2 times more *Culicoides* were found on a cow than on a Shetland pony (Elbers & Meiswinkel, 2015). However, this could be variable between species and size of horses/cows. There is a lot of uncertainty around this parameter; therefore, the range of values considered during the sensitivity analysis was large.

Some parameters such as the length of the host and vector latent periods (1/*ε* and 1/*ν*, respectively) and the case fatality (*m_H_*) cannot be influenced by control measures. To reduce the infectious period of hosts, infected hosts are often euthanized. Analysis of the studies from the systematic search showed that clinical signs appear a mean of 0.8 days after onset of viraemia. Euthanasia would only take place after clinical signs are observed (which is likely to be very variable), a vet responds, and makes a diagnosis. Therefore, at the time of euthanasia, a proportion of the infectious period is likely to have already passed. Results show that the length of the infectious period does not influence the duration of the outbreak as much as the vector:host ratio. This suggests that vector control may be a more efficient method of controlling outbreaks. Vector control reduces the life‐span of the midges (1/μ*_V_*) and therefore the probability they complete the extrinsic incubation period and become infectious is also reduced. There are several methods of reducing the bite rate of vectors (1/a); these include insect repellents, fly rugs, stabling horses during times of high midge activity and insect‐proofing stable areas (Barba et al., 2019; Meiswinkel et al., 2000; Robin et al., 2015). Vaccination to prevent the horse from becoming infected reduces (to 0 in a perfect vaccine) the transmission probability from vector to host (*p_V_*). The transmission probability from hosts to vectors (*p_H_*) would also likely be reduced if a partially immune animal became infected due to lower levels of viraemia (Scanlen et al., 2002).

Euthanasia of infected horses for ethical reasons affects our ability to assess the case fatality. However, as mentioned previously, during an outbreak it is likely that equids with severe clinical signs that are unlikely to survive would be euthanized. Therefore, including these euthanized hosts when considering those that do not survive would not be unrealistic in the model. The longer infectious period of surviving hosts may also be informative for policy makers when deciding if culling should be used as a control strategy. The rapid onset of death in AHSV‐infected naive horses is itself thought to limit virus spread. The persistence (long term) of AHSV in the field is thought to be heavily dependent on the availability and distribution of alternative, possibly asymptomatic hosts, such as zebras or potentially donkeys or mules. Therefore, it is important to consider secondary hosts and their ability to aid spread of AHSV between premises, even if they are not specifically included in the model.

Overall, the re‐parameterization of the model is more informed by the literature. Robin et al. (2016) stated that ‘extensive further research is required if the equine industry is to avoid or effectively contain an AHS epizootic in disease‐free regions’. Here, four key areas for further research were highlighted. These included improving the accuracy of disease modelling, which we have aimed to address in this study. This study also supports the importance of further research on the vector competence of certain *Culicoides* species and our knowledge of their distribution due to the lack of robust literature to parameterize these aspects of the model. Other areas highlighted included methods of reducing transmission, such as vaccination and methods of reducing vector bite rates. Sensitivity analysis highlighted the importance of vector control, supporting the suggestion in Robin et al. (2016) that developing more effective and practical methods of preventing *Culicoides* blood‐feeding on horses may be key in AHSV control.

## CONFLICT OF INTEREST

The authors declare no conflict of interest.

## ETHICS STATEMENT

The authors confirm that the ethical policies of the journal, as noted on the journal's author guidelines page, have been adhered to. No ethical approval was required.

## Supporting information

Supporting Information

## Data Availability

All data used in this manuscript are available from the references cited.
