# Supplementary material for: Re‐parameterization of a mathematical model of African horse sickness virus using data from a systematic literature search
Source: Transbound Emerg Dis. 2022 Jan 12;69(4):e671–81. doi: 10.1111/tbed.14420 (PMC9543668; doi:10.1111/tbed.14420)
Supplement: Supplementary file 1 — Supporting Information [file TBED-69-e671-s001.docx]

Re-parameterisation of a mathematical model of African horse sickness virus using data from a systematic literature search

Emma L. Fairbanks1, Marnie L. Brennan1, Peter P.C. Mertens1, Michael J. Tildesley2, and Janet

M. Daly1

1School of Veterinary Medicine and Science, University of Nottingham, Loughborough. LE12 5RD, UK 2The Zeeman Institute for Systems Biology & Infectious Disease Epidemiology Research, School of Life Sciences and Mathematics Institute, University of Warwick, Coventry, CV4 7AL, UK

# Supplementary file 1: Updated model parameters

Table [S1](#_bookmark0) details the vector parameters previously used by [Backer & Nodelijk (2011)](#_bookmark8) and the modified values from the literature. Unchanged parameters are also given in this table.

Table S1: Updated model parameters from other literature. The temperature-dependent rates are calculated for 18°C.

| Parameter | Symbol | Previous value | | Updated value* | |
| --- | --- | --- | --- | --- | --- |
|  |  | Default value | 5–95% range | Default value | 5–95% range |
| Blood feeding interval | 1/a | 7.5 | 4.7–17.7 | 6.3^1^ |  |
| EIP | 1/ν | 16 | 9.2–48 | 10.9^2^ |  |
| No. stages | k | 10 |  |  |  |
| Vector life-span | 1/µ*V* | 22 | 16–31 | 11.9^3^ |  |
| Transmission probability host to vector | p*H* | 0.04 | 0.01–0.1 | 0.52^2^ | 0.45–0.59^2^ |
| Transmission probability vec­tor to host | p*V* | 0.77 | 0.5–0.95 |  |  |
| Vector:host ratio | ρ | 226 | 1–4219 |  |  |
| Initial number of hosts | N*H* | 66 | 32–100 |  |  |

Time periods are given in days. The parameter values used in [Backer & Nodelijk (2011](#_bookmark8)) are described in the previous value column and the parameter values used in our model are described by the updated value column. Where the updated column is blank no new information on these parameters was found. * 1 = updated using [Mullens et al. (200](#_bookmark32)4), 2 = updated using [Carpenter et al. (2011),](#_bookmark10) 3 = updated using [Gerry & Mullens (2000).](#_bookmark14)

The temperature-dependent rates are calculated for 18°C, to represent the average temperature of equine dense areas in the UK in August. At this time, when there is a seasonal increased abundance of midges, in such areas, with large horse populations, is when an outbreak of African horse sickness virus is likely to cause the most impact. When considering an outbreak in a different climate these parameters may need to be adjusted.

There are limited data available for the temperature dependent parameters affecting the transmission of AHSV. For example, an initial search of PubMed using the terms ‘African horse sickness’ AND ‘extrinsic incubation periods’ yielded only five results. Of these, two were studies on different viruses (bluetongue virus and equine encephalosis virus (v[an der Saag](#_bookmark42) [et al., 2017;](#_bookmark42) [Venter et al., 19](#_bookmark44)99)). [Carpenter et al. (2011),](#_bookmark10) published shortly after Backer and Nodelijk published their model in 2011, reanalysed data from [Wittmann et al. (2002)](#_bookmark50) (which was also found in the search) using novel statistical methodology. The fifth search result [(Sánchez-Matamoros et al., 2016)](#_bookmark38) was a mathematical modelling study that referenced [Carpenter et al. (2011).](#_bookmark10) [Carpenter et al. (2011)](#_bookmark10) derived the extrinsic incubation period (EIP) and probability of transmission from host to vector from AHSV-4 infection experiments in *Culicoides sonorensis*.

In an attempt to find additional articles quantifying the probability of transmission from host to vector, the search terms ‘African horse sickness’ AND (‘vector competence’ OR ‘susceptibility’) were used to search PubMed. After screening the titles and abstracts, apart from the original [Wittmann et al.](#_bookmark50) [(200](#_bookmark50)2) article and the [Carpenter et al.](#_bookmark10) [(2011)](#_bookmark10) article reanalyzing the data, four other articles were found (V[enter et al., 2000,](#_bookmark43) [2009,](#_bookmark47) [2010;](#_bookmark46) [Venter & Pawesk](#_bookmark45)a, [2007).](#_bookmark45) These articles all compare the susceptibility of African *Culicoides* species to AHSV. Findings from these studies are highly variable between species (V[enter et al.,](#_bookmark43) [2000,](#_bookmark43) [2009;](#_bookmark47) [Venter & Paweska, 2007),](#_bookmark45) populations (V[enter et al., 2009),](#_bookmark47) serotypes (V[enter et al., 2009,](#_bookmark47) [2010),](#_bookmark46) isolates (V[enter et al., 2009,](#_bookmark47) [2010;](#_bookmark46) [Venter & Pawesk](#_bookmark45)a, [2007)](#_bookmark45) and seasons (V[enter et al.,](#_bookmark47) [2009).](#_bookmark47) The species of *Culicoides* with the estimated largest probability of being infectious within 10 days at 23.5°C of a viraemic blood meal and this probability in these studies by serotype are given in Table [S2.](#_bookmark1) In this study, we use the parameter suggested by [Carpenter](#_bookmark10) [et al.](#_bookmark10) [(2011)](#_bookmark10) (0.52) as it was parameterised with the EIP used in the model and for the same species as other parameters used in the model. The model is also simulated for a temperature of 18°C, which is within the span of temperatures examined in the study (15–30°C).

This trend of limited data availability for the dynamics of AHSV and *Culicoides* continued in the search for other parameters. Other *Culicoides*-borne disease models parameterised the blood feeding interval, assumed to be the time between blood feeding and oviposition, function derived by [Mullens et al. (2004)](#_bookmark32) for *C. sonorensis* [(Gubbins et al., 2008,](#_bookmark15) [2014;](#_bookmark16) [Haider](#_bookmark18) [et al., 2019).](#_bookmark18) The updated vector lifespan used was derived by [Gerry & Mullens (2000)](#_bookmark14) who attempted to quantify how the expected lifespan of a *C. sonorensis* varies according to temperature using data from a dairy farm in California, USA. The Backer and Nodelijk paper (2011) mentioned this study but suggested that the mortality for midges in the field was significantly higher than that seen in lab experiments by [Wittmann et al. (20](#_bookmark50)02), the value they used in their model. This leads to reduced transmission when the [Gerry & Mullens](#_bookmark14) [(2000)](#_bookmark14) parameters are used However, the midge mortality rate may be higher in the field due to environmental effects. The PubMed search ‘Culicoides’ AND (‘survival’ OR ‘mortality’) did not identify any other studies of interest.

Table S2: Species of Culicoides with the largest probability of being infected within 10 days at 23.5 °C of a viraemic blood meal.

| Serotype | *Culicoides* species | Probability | Reference |
| --- | --- | --- | --- |
| AHSV-1 | C. *leucostictus* | 0.08 | [Venter et al. (2010)](#_bookmark46) |
| AHSV-2 | C. *leucostictus* | 0.38 | [Venter et al. (2010)](#_bookmark46) |
| AHSV-3 | C. *bolitinos* | 0.17 | [Venter et al. (2009)](#_bookmark47) |
| AHSV-4 | C. *imicola* | 0.05 | [Venter et al. (2010)](#_bookmark46) |
| AHSV-5 | C. *bolitinos* | 0.21 | [Venter et al. (2000)](#_bookmark43) |
| AHSV-6 | C. *imicola* | 0.41 | [Venter et al. (2009)](#_bookmark47) |
| AHSV-7 | C. *imicola* | 0.33 | [Venter et al. (2009)](#_bookmark47) |
| AHSV-8 | C. *imicola* | 0.27 | [Venter et al. (2000)](#_bookmark43) |
| AHSV-9 | C. *zulluensis* | 0.20 | [Venter et al. (2009)](#_bookmark47) |

# Supplementary file 2: Mathematical model description

Let *S_H_*(*t*), *E_H_*(*t*), *I_H_*(*t*) and *R_H_*(*t*) denote the number of susceptible, latent, infectious and recovered horses. We also denote the total number of alive horses as *N_H_*. The recovered class is not considered in vector populations; this is because of their short lifespans; therefore, we denote *S_V_* (*t*), *E_V_* (*t*) and *I_V_* (*t*) as the number of susceptible, latent and infectious vectors. In the model, vector mortality and birth also occur at an equal rate, *µ_V_*, which is dependent on temperature; this allows us to consider the total number of vectors as a constant, denoted *N_V_* , determined by the vector:host ratio. We will assume that all vectors are born susceptible.

The rate at which new infections occur depends on the bite rate of midges, the transmission probabilities and the number of individuals in the *S_H_*, *I_H_*, *S_V_* and *I_V_* classes. The bite rate (1*/*$\epsilon$) depends on temperature. The transmission probabilities from host-to-vector and vector-to-host, *p_H_* and *p_V_* , respectively, is the probability that an infectious bite results in a susceptible individual becoming infected. The infection rates for susceptible hosts and vectors are therefore given by

and

where *N_V_ /N_H_* is the vector to host ratio (*ρ*), *I_H_/N_H_* is the proportion of hosts infected and

*I_V_ /N_V_* is the proportion of vectors infected.

The rate latent midges become infected is dependent on temperature and given by the extrinsic incubation period (EIP): 1*/υ*. The simplest way to introduce recovery is to assume the infected host recovers at a constant rate *γ*, which is the inverse of their infectious period.

The adapted deterministic ODE system is given by:

where the nature of the parameters is described in Tables 5 and S1. The overall recovery rate from the first and second infectious classes are calculated as

*γ*^1^ = 1

*T*

and *γ*^2^ = 1

*,* (16)

respectively.

*T*

*− T*

1

*inf*

2

*inf*

1

*inf*

The latent period of hosts, infectious period of hosts, and EIP of vectors are divided into multiple stages; this allows them to have a gamma distribution. Here, for example the latent stage (*E_H_*) is subdivided into *i* stages; each with mean transition rate *c/i*, where *c* is the transition rate from stage *E_H_* to stage *I*^1^. Using this method, *E_H_*(*t*) is replaced by a series

*H*

of *i* stages *E*^1^

*H,*1

(*t*), *E*^1^

(*t*), *. . . ,*E1

(*t*). When individuals first transition from the previous

stage, the individuals will enter the first stage *E*^1^ (*t*). Then, once each stage is completed,

*H,*2

*H,i*

*H,*1

they will continue to pass through all stages successively until they complete the *E*^1^

*H,i*

(*t*)

stage.

# Supplementary file 3: Sensitivity analysis

The Latin hypercube sampling partial rank correlation coefficient method (LHS-PRCC method) was used for the sensitivity analysis. This method was described in [Blower &](#_bookmark9) [Dowlatabadi (1994).](#_bookmark9) If the PRCC is close to 0, the parameter is weakly (or not at all) correlated with the output parameter. As the PRCC approaches 1 or -1 it represents a positive or negative correlation, respectively, with 1 and -1 reflecting a perfect correlation. This method can also be applied to each time-step in order to see the sensitivity of the parameters over the time-course; here we refer to this as the heat map method.

We perform the calculation of the PRCC 100 times for 100 sets of parameters each time. The median PRCC and its variation are examined using a box-plot. The duration of the outbreak is considered to be when it is *<* 10^-3^ from its final steady state. This is due to the numerical methods resulting in minute changes in the populations as the steady state is approached. For the heat map method, the PRCC is calculated only once per time-step and is performed 100 times for 100 parameters sets; the value plotted is the mean PRCC of the outputs. The heat maps will vary from white to red for 0 to 1 and from white to blue from 0 to -1. Therefore the more positively correlated a parameter is the more red it will be, and the more negatively correlated a parameter is the more blue it will be. If a result was not found to be significant through the Student’s t-test (*p >* 0*.*05) it was coloured white. Matlab was used for calculations and to produce figures, LHS was performed using the *lhsdesign* function. Here the ODE solver *ode23s* was used, alternatively to *ode45*, to resolve stiffness issues for some sampled parameter sets.

Parameters were all selected for a uniform distribution (upper and lower bounds shown in Table [S3).](#_bookmark3) The upper and lower bounds for the latent and infectious periods of hosts were set to the maximum and minimum values found in the systematic review. However, this caused numerical issues when the length of the infectious period selected for dying hosts was longer than that of surviving hosts. Therefore, the infectious period of surviving hosts was set to the infectious period of the dying hosts multiplied by 1.2, as the mean infectious period of surviving hosts found in the systematic review was 1.2 times longer than that of dying hosts. The transmission probability from host to vector was set to the 5–95% range found in [Carpenter et al. (2011).](#_bookmark10) As the parameters for the transmission probability from vector to host, vector to host ratio and the initial number of hosts were not updated, the 5–95% range from Backer and Nodelijk (2011) were used. In Backer and Nodelijk (2011) the host case fatality 5–95% range varied -/+0.27 from the default value. This variance was also applied to the updated case fatality with a maximum of 1. No confidence intervals were given for the blood-feeding interval and vector lifespan therefore the upper and lower bounds were set to +/-25% of the updated default values. The extrinsic incubation period is calculated using two parameters estimated in Carpenter *at al*. (2011) where the 5–95% confidence intervals are given. In order to find the lower and upper bound for the sensitivity analysis the extrinsic incubation period was calculated for 10000 random values between the 5–95% confidence intervals for these parameters. The upper and lower bounds are then taken from 5% and 95% of these 10000 values, respectively.


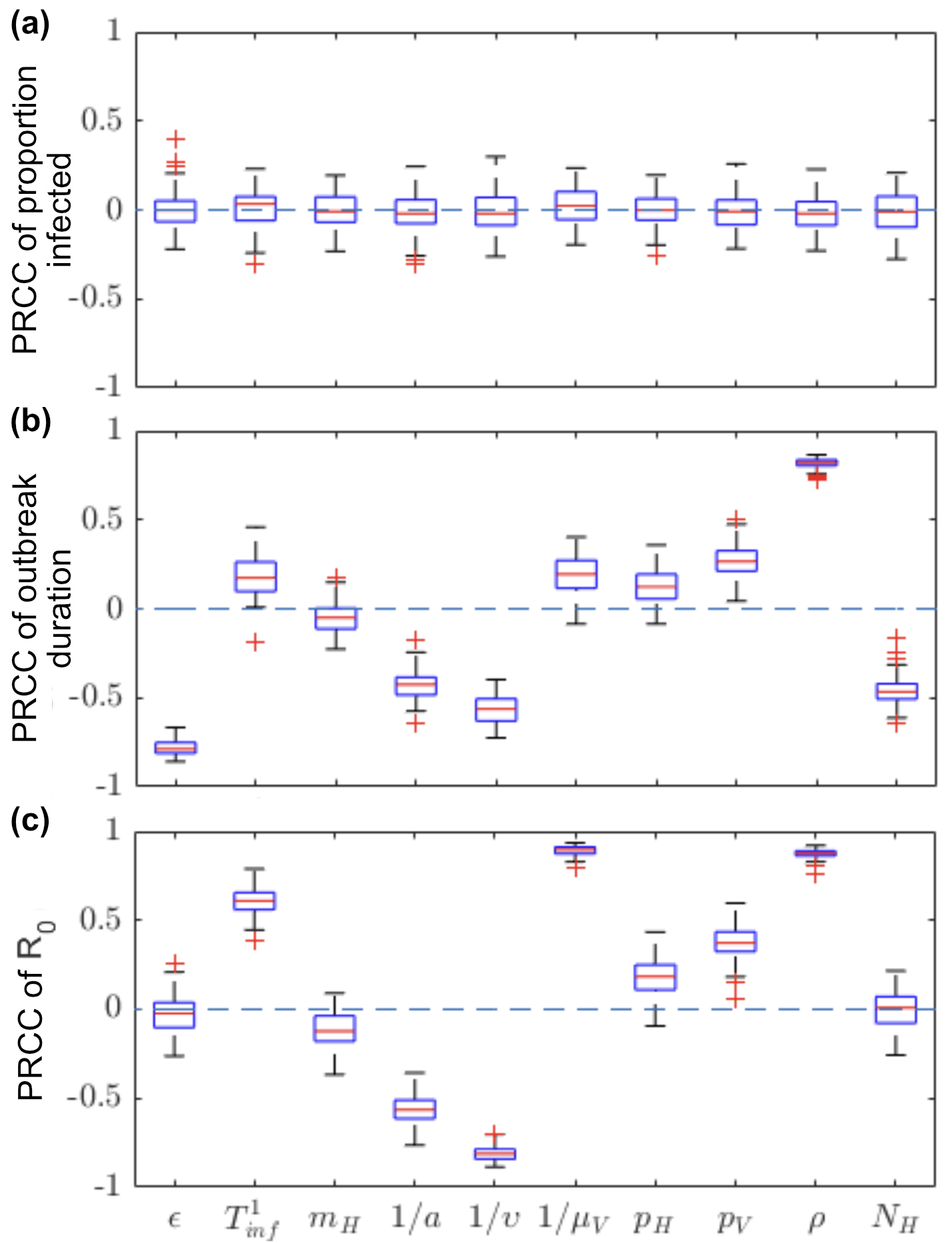


Figure S1: Box and whisker plots of PRCC between the model parameters and the (a) proportion of hosts infected, (b) outbreak duration and (c) *R*_0_. On each box, the central mark indicates the median, and the bottom and top edges of the box indicate the 25*^th^* and 75*^th^* percentiles, respectively. The whiskers extend to the most extreme data points not considered outliers, and the outliers are plotted individually using the + symbol.

Table S3: The upper and lower bounds of parameters used for the sensitivity analysis. Time

periods are given in days. To avoid numerical errors *T* ^2^ = 1*.*2*T* ^1^ .

*inf*

*inf*

| Parameter | Lower bound | Upper bound |
| --- | --- | --- |
| 1*/*$\epsilon$ | 2 | 11 |
| *T* ^1^ 2 7  *inf* | | |
| *m_H_* | 0.57 | 1 |
| 1*/a* | 4.7 | 7.9 |
| 1*/υ* | 8.6 | 13.5 |
| 1*/µ_V_* | 8.9 | 14.9 |
| *p_H_* | 0.45 | 0.59 |
| *_V_* | 0.5 | 0.95 |
| *ρ* | 1 | 4219 |
| *N_H_* | 32 | 100 |

The PRCC method is applied to the duration of the outbreak, total equine infections over the duration of the outbreak and *R*_0_ (Figure [S1).](#_bookmark2) Here *R*_0_ is calculated using the equation suggested by [Backer & Nodelijk (2011);](#_bookmark8) given as

The heat map PRCC method on the case incidences in each time-step are shown in Figure [S2.](#_bookmark4) Table [S4](#_bookmark5) also shows the median PRCC values and significance for the PRCC on the duration of the outbreak, total number of equines infected and *R*_0_. Here two-tailed Student’s t-tests were used to determine the p-values.

We observe from Figure [S1](#_bookmark2) and Table [S4](#_bookmark5) that none of the parameters significantly affect the proportion of equids infected. Despite the uncertainty in many parameters, most simulations in the sensitivity analysis resulted in all the equids on a premises becoming infected. The duration of the latent period (1*/*$\epsilon$) and number of horses on the premises (*N_H_*) are not used in the calculation of *R*_0_, therefore they do not significantly influence its value. The host case fatality (*m_H_*) did not significantly influence *R*_0_, suggesting that culling may not be an effective control strategy. The parameter which most significantly influences the duration of the outbreak is the vector:host ratio. This is associated with shorter outbreaks. This can also be observed in the heat map (Figure [S2).](#_bookmark4) The heat map shows that longer host latent periods (1*/*$\epsilon$), duration between vector bites (1*/a*) and extrinsic incubation periods (1*/υ*) increase the duration of the outbreak (negatively associated with case incidence during the early stages). However, the host infectious period (*T_inf_* ), vector life-span (1*/µ_V_* ), host


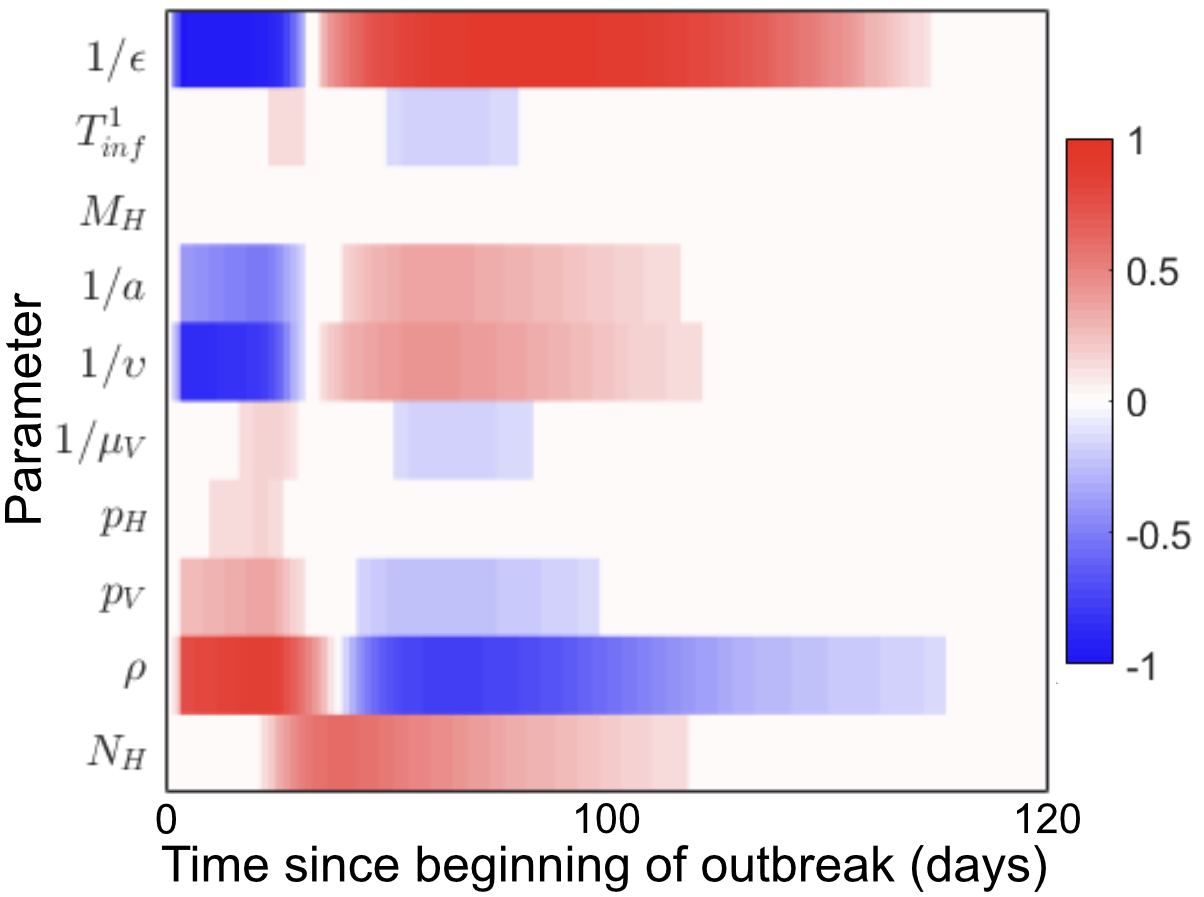


Figure S2: Heatmap of the PRCC of case incidence each timestep.

Table S4: Results from the sensitivity analysis ; the PRCC values and t-values of parameters on the proportion of equines infected, duration of the outbreak and *R*_0_. The PRCC values are given to 3 decimal places. The t-values are given to 2 decimal places. P-values where calculated using a two-tailed students t-test with 89 degrees of freedom. * *p ≤* 0*.*05, ** *p* *≤* 0*.*01 and *** *p ≤* 0*.*001.

| PRCC of: Proportion infected Outbreak duration *R*_0_ | | | | | | |
| --- | --- | --- | --- | --- | --- | --- |
| Parameter | Mean | t value | Mean | t value | Mean | t value |
| 1*/*$\epsilon$ | -0.000 | -0.00 | -0.778 | -5*.*78*** | -0.022 | -0.22 |
| *T* 1 | 0.007 | 0.07 | 0.180 | 1.97 | 0.609 | 9*.*64*** |
| *m_H_* | -0.001 | -0.01 | -0.053 | -0.51 | -0.121 | -1.13 |
| 1*/a* | -0.021 | -0.21 | -0.429 | -3*.*55*** | -0.558 | -4*.*43*** |
| 1*/υ* | -0.010 | -0.10 | -0.565 | -4*.*47*** | -0.810 | -5*.*96*** |
| 1*/µ_V_* | 0.027 | 0.27 | 0.188 | 2*.*07* | 0.892 | 26*.*91*** |
| *p_H_* | -0.000 | -0.00 | 0.124 | 1.31 | 0.184 | 2*.*01* |
| *p_V_* | -0.010 | -0.10 | 0.270 | 3*.*12** | 0.377 | 4*.*73*** |
| *ρ* | -0.020 | -0.19 | 0.817 | 18*.*88*** | 0.875 | 24*.*53*** |
| *N_H_* | -0.010 | -0.10 | -0.461 | -3*.*78*** | -0.001 | -0.01 |

*inf*

to vector (*p_H_*) and vector to host (*p_V_*) transmission rates, and the vector:host ratio are associated with outbreaks that spread more rapidly upon emergence (positively associated with case incidence for up to approximately the first 40 days).

# Supplementary table S5

Table S5: Qualitative synthesis of the studies found reporting AHSV experimental infection in a naive equid. If reported the time until viraemia, onset of clinical signs and death of the equid is given. Where possible the duration of viraemia and clinical signs is also given. *n/a = survived; †= died; ‡= euthanised; †/‡= died or euthanised. = data unavailable.

[Lelli et al. (2013)](#_bookmark24) Intravenous PCR/isolation AHSV-9 Horse 11/12 10 n/a 4 5

Reference

Inoculation route

Viraemia detection method

Days to: Duration of:

Serotype Equid sp. Viraemia Clinical Death* Viraemia Clinical

signs

signs

Horse 7/8 8 n/a 5 3

[Alberca et al.](#_bookmark6) [(2014)](#_bookmark6)

[Lulla et al. (2017)](#_bookmark25) Intravenous PCR AHSV-4 Horse 4 6 10‡ 6 4

[von Teichman et al.](#_bookmark48) [(2010)](#_bookmark48)

[Guthrie et al.](#_bookmark17) [(2009)](#_bookmark17)

[Scanlen](#_bookmark39) [(2002)](#_bookmark39)

[et](#_bookmark39)

[al.](#_bookmark39) Subcutaneous Isolation

AHSV-5

Horse

3

4

7†

4

3

Horse 4 4 8† 4 4

Intravenous PCR/isolation AHSV-9 Horse 3/3 3 5† 2 2

Horse 3/3 3 6† 3 3

Horse 3/3 3 6† 3 3

Intravenous Isolation AHSV-5 Horse 3 ‡

AHSV-6 Horse 5 ‡

AHSV-8 Horse 5 ‡

AHSV-9 Horse 5 ‡ Intravenous PCR/isolation AHSV-4 Horse 8/8 n/a

[Du Plessis et al.](#_bookmark12) [(1998)](#_bookmark12)

Subcutaneous n/a AHSV-5 Horse 7‡

[Roy et al. (1996](#_bookmark37)) Isolation AHSV-4 Horse 6 6 10† 4 4

| [Martínez-](#_bookmark26) [Torrecuadrada](#_bookmark26) [et al. (1996)](#_bookmark26) | Intravenous | Isolation | AHSV-4 | Horse | 6 |  | † |  | |
| --- | --- | --- | --- | --- | --- | --- | --- | --- | --- |
| [Stone-Marschat et](#_bookmark40) | Intravenous | Isolation | AHSV-4 | Horse | 3 | 4 | †/‡ |  |  |
| [al. (1996)](#_bookmark40) |  |  |  |  |  |  |  |  |  |
|  |  |  |  | Horse | 3 | 4 | †/‡ |  |  |
|  |  |  |  | Horse | 3 | 4 | †/‡ |  |  |
|  |  |  |  | Horse | 3 | 4 | †/‡ |  |  |
| [J. House et al.](#_bookmark22) | Intravenous | Isolation | AHSV-4 | Horse | 3 | 5 | 7† | 4 | 2 |
| [(1994)](#_bookmark22) |  |  |  |  |  |  |  |  |  |
|  |  |  |  | Horse | 4 | 6 | 8† | 4 | 2 |
| [Hassanain (1992)](#_bookmark19) |  | n/a | AHSV-9 | Horse |  |  | 13† |  |  |

[Mirchamsy &](#_bookmark31) [Taslimi (1968)](#_bookmark31)

[Ozawa & Bahrami](#_bookmark33) Intravenous [(1966)](#_bookmark33)

n/a

AHSV-9

Horse

8

9†

Intravenous n/a AHSV-9 Horse 17†

[C. House et al.](#_bookmark21) [(1990)](#_bookmark21)

[El Hasnaoui et al.](#_bookmark13) [(1998)](#_bookmark13)

[van Rijn et al.](#_bookmark41) [(2018)](#_bookmark41)

Subcutaneous n/a AHSV-1 Horse †/‡

AHSV-2 Horse †/‡

AHSV-3 Horse †/‡

AHSV-4 Horse †/‡

Subcutaneous n/a AHSV-4 Donkey

Donkey Donkey Donkey Mule Mule Mule

Intravenous PCR AHSV-5 Horse 2 3 6† 4 3

Horse 3 4 8† 5 4

[Martínez-](#_bookmark27) [Torrecuadrada](#_bookmark27) [et al. (1997)](#_bookmark27)

[Minke et al. (2012)](#_bookmark28) Intravenous PCR AHSV-4 Horse 8 n/a

[Alexander &](#_bookmark7) [Du Toit (1934)](#_bookmark7) [Dubourget et al.](#_bookmark11) [(1992)](#_bookmark11)

[Mirchamsy &](#_bookmark30) [Taslimi (1964b)](#_bookmark30)

[J. House et al.](#_bookmark23) [(1992)](#_bookmark23)

[Hazrati & Ozawa](#_bookmark20) [(1965)](#_bookmark20)

Intravenous n/a AHSV-4 Horse 9†

Horse 16†

Horse n/a

Intravenous n/a Horse 3 6† 3

Subcutaneous Isolation AHSV-4 Horse 2 9† 7

Horse 6 n/a 5

Intravenous n/a AHSV-9 Horse 12†

Intravenous n/a AHSV-9 Horse 7†

Horse 7†

Horse 7†

Intravenous n/a ASHV-9 Horse 14†

Horse n/a

Donkey n/a

[Whitworth (1930)](#_bookmark49) n/a Horse 3 5 ‡ 2

[Quan et al. (2010)](#_bookmark36) Intravenous PCR AHSV-4 Horse 7

[Mirchamsy &](#_bookmark29) [Taslimi (1964a)](#_bookmark29)

Intravenous n/a AHSV-9 Horse †

Horse †

Horse †

Horse †

[Ozawa et al. (1970)](#_bookmark34) n/a AHSV-9 Horse 9†

[Ozawa et al. (1965)](#_bookmark35) Intravenous n/a AHSV-9 Horse 14†

# Supplementary table S6

Results from the Kruskal-Wallis test to determine differences between the time until viraemia, onset of clinical signs and death of different serotypes of African horse sickness virus and the method used for their inoculation.

Table S6: The chi-squared value, degrees of freedom and p-values are given. Serotypes with only one data value are not included.

Time until:

*χ*2

df

p-value

*†*

| Serotype |  | | |
| --- | --- | --- | --- |
| Viraemia | 4.58 | 4 | 0.33 |
| Viraemia *†* | 3.68 | 2 | 0.16 |
| Clinical signs | 2.09 | 2 | 0.35 |
| Death | 2.68 | 2 | 0.26 |
| Inoculation method | | | |
| Viraemia | 0.55 | 1 | 0.46 |
| Clinical signs | 0.08 | 1 | 0.78 |
| Death | 0 | 1 | 1 |

# References

Alberca, B., Bachanek-Bankowska, K., Cabana, M., Calvo-Pinilla, E., Viaplana, E., Frost, L., . . . Castillo-Olivares, J. (2014). Vaccination of horses with a recombinant modified vaccinia Ankara virus (MVA) expressing African horse sickness (AHS) virus major capsid protein VP2 provides complete clinical protection against challenge. *Vaccine*, *32* (29), 3670–3674. doi: 10.1016/j.vaccine.2014.04.036

Alexander, R. A., & Du Toit, P. J. (1934). The immunization of horses and mules against horse sickness by means of the neurotropic virus of mice and guinea pigs. *Onderstepoort J Vet Res*, *2* , 375-391.

Backer, J. A., & Nodelijk, G. (2011). Transmission and control of African horse sickness in The Netherlands: a model analysis. *PLoS One*, *6* (8), e23066. doi: 10.1371/journal.pone

.0023066

Blower, S. M., & Dowlatabadi, H. (1994). Sensitivity and uncertainty analysis of complex models of disease transmission: an HIV model, as an example. *Int Stat Rev*, 229-243. doi: 10.2307/1403510

Carpenter, S., Wilson, A., Barber, J., Veronesi, E., Mellor, P., Venter, G., & Gubbins,

S. (2011). Temperature dependence of the extrinsic incubation period of orbiviruses in Culicoides biting midges. *PloS one*, *6* (11), e27987. doi: 10.1371/journal.pone.0027987

Dubourget, P., Preaud, J., Detraz, N., Lacoste, F., Fabry, A., Erasmus, B., & Lombard, M. (1992). Development, production and quality control of an industrial inactivated vaccine against African horse sickness virus serotype 4. *Bluetongue, African horse sickness and related orbiviruses, CRC Press, Boca Raton*, 874-886.

Du Plessis, M., Cloete, M., Aitchison, H., & Van Dijk, A. (1998). Protein aggregation complicates the development of baculovirus-expressed African horse sickness virus serotype 5 VP2 subunit vaccines. *Onderstepoort J Vet Res*, *65* (4), 321-329.

El Hasnaoui, H., El Harrak, M., Zientara, S., Laviada, M., & Hamblin, C. (1998). Serological and virological responses in mules and donkeys following inoculation with African horse sickness virus serotype 4. *Arch Virol Suppl*, *14*, 29-36. doi: 10.1007/978-3-7091-6823-3\ underline{}4

Gerry, A. C., & Mullens, B. A. (2000). Seasonal abundance and survivorship of *Culicoides* *sonorensis* (*Diptera*: *Ceratopogonidae*) at a southern California dairy, with reference to potential bluetongue virus transmission and persistence. *J Med Entomol*, *37* (5), 675-688. doi: 10.1603/0022-2585-37.5.675

Gubbins, S., Carpenter, S., Baylis, M., Wood, J. L., & Mellor, P. S. (2008). Assessing the risk of bluetongue to UK livestock: uncertainty and sensitivity analyses of a temperature- dependent model for the basic reproduction number. *J R Soc Interface*, *5* (20), 363-371. doi: 10.1098/rsif.2007.1110

Gubbins, S., Turner, J., Baylis, M., Van der Stede, Y., van Schaik, G., Abrahantes, J. C., & Wilson, A. J. (2014). Inferences about the transmission of Schmallenberg virus within and between farms. *Prev Vet Med* , *116* (4), 380-390. doi: 10.1016/j.prevetmed.2014.04.011

Guthrie, A. J., Quan, M., Lourens, C. W., Audonnet, J.-C., Minke, J. M., Yao, J.,

. . . MacLachlan, N. J. (2009). Protective immunization of horses with a recombi- nant canarypox virus vectored vaccine co-expressing genes encoding the outer capsid proteins of African horse sickness virus. *Vaccine*, *27* (33), 4434-4438. doi: 10.1016/ j.vaccine.2009.05.044

Haider, N., Kjær, L. J., Skovgård, H., Nielsen, S. A., & Bødker, R. (2019). Quantifying the potential for bluetongue virus transmission in danish cattle farms. *Sci Rep*, *9* (1), 1–12. doi: 10.1038/s41598-019-49866-8

Hassanain, M. M. (1992). Preliminary findings for an inactivated African horse sickness vaccine using binary ethyleneimine. *Rev Elev Med Vet Pays Trop*, *45* (3-4), 231-234.

Hazrati, A., & Ozawa, Y. (1965). Monovalent live-virus horse-sickness vaccine. *Bull Off Int Epizoot* , *64* , 683-695.

House, C., Mikiciuk, P. E., & Beminger, M. L. (1990). Laboratory diagnosis of African horse sickness: comparison of serological techniques and evaluation of storage meth- ods of samples for virus isolation. *J Vet Diagn Invest*, *2* (1), 44-50. doi: 10.1177/ 104063879000200108

House, J., Lombard, M., Dubourget, P., House, C., & Mebus, C. A. (1994). Further studies on the efficacy of an inactivated African horse sickness serotype 4 vaccine. *Vaccine*, *12* (2), 142-144. doi: 10.1016/0264-410x(94)90052-3

House, J., Lombard, M., House, C., Dubourget, P., & Mebus, C. A. (1992). Efficacy of an inactivated vaccine for African horse sickness serotype 4. *Bluetongue, African horse sickness and related orbiviruses, CRC Press, Boca Raton*, 891-895.

Lelli, R., Molini, U., Ronchi, G. F., Rossi, E., Franchi, P., Ulisse, S., . . . others (2013). Inactivated and adjuvanted vaccine for the control of the African horse sickness virus serotype 9 infection: Evaluation of efficacy in horses and guinea-pig model. *Vet Ital* , *49* (1), 89-98.

Lulla, V., Losada, A., Lecollinet, S., Kerviel, A., Lilin, T., Sailleau, C., . . . Roy, P. (2017). Protective efficacy of multivalent replication-abortive vaccine strains in horses against African horse sickness virus challenge. *Vaccine*, *35* (33), 4262-4269. doi: 10.1016/j.vaccine

.2017.06.023

Martínez-Torrecuadrada, J. L., Díaz-Laviada, M., Roy, P., Sánchez, C., Vela, C., Sánchez- Vizcaíno, J. M., & Casal, J. I. (1996). Full protection against African horsesickness (AHS) in horses induced by baculovirus-derived AHS virus serotype 4 VP2, VP5 and VP7. *J Gen Virol* , *77* (6), 1211-1221. doi: 10.1099/0022-1317-77-6-1211

Martínez-Torrecuadrada, J. L., Díaz-Laviada, M., Roy, P., Sánchez, C., Vela, C., Sanchez- Vizcaino, J. M., & Casal, J. I. (1997). Serologic markers in early stages of African horse sickness virus infection. *J Clin Microbiol* , *35* (2), 531-535. doi: 10.1128/jcm.35.2.531-535

.1997

Minke, J. M., Audonnet, J.-C., Guthrie, A. J., MacLachlan, N. J., & Yao, J. (2012). *Vaccine against African horse sickness virus.* Google Patents. (US Patent 8,168,200)

Mirchamsy, H., & Taslimi, H. (1964a). Attempts to vaccinate foals with living tissue culture adapted horse sickness virus. *Arch Inst Razi* , *17* (1), 17-27. doi: 10.22092/ari.1965.108547

Mirchamsy, H., & Taslimi, H. (1964b). Immunization against African horse-sickness with tissue culture adapted neurotropic viruses. *Brit vet J*, *120* (10), 481-486. doi: 10.1016/ S0007-1935(17)41556-9

Mirchamsy, H., & Taslimi, H. (1968). Inactivated African horse sickness virus cell culture vaccine. *Immunology* , *14* (1), 81-88.

Mullens, B., Gerry, A., Lysyk, T., & Schmidtmann, E. (2004). Environmental effects on vector competence and virogenesis of bluetongue virus in *Culicoides*: interpreting laboratory data in a field context. *Vet Ital* , *40* (3), 160-166.

Ozawa, Y., & Bahrami, S. (1966). African horse-sickness killed-virus tissue culture vaccine.

*Can J Comp Med Vet Sci* , *30* (11), 311-314.

Ozawa, Y., Hazrati, A., & Bahrami, S. (1970). African horse-sickness live and killed virus tissue culture vaccine. *Arch Inst Razi* , *22* , 103-111.

Ozawa, Y., Hazrati, A., & Erol, N. (1965). African horse-sickness live-virus tissue culture vaccine. *Arch Inst Razi* , *18* , 61-84.

Quan, M., Lourens, C. W., MacLachlan, N. J., Gardner, I. A., & Guthrie, A. J. (2010). Development and optimisation of a duplex real-time reverse transcription quantitative PCR assay targeting the VP7 and NS2 genes of African horse sickness virus. *J Virol Methods*, *167* (1), 45-52. doi: 10.1016/j.jviromet.2010.03.009

Roy, P., Bishop, D. H., Howard, S., Aitchison, H., & Erasmus, B. (1996). Recombinant baculovirus-synthesized African horse sickness virus (AHSV) outer-capsid protein VP2 provides protection against virulent AHSV challenge. *J Gen Virol*, *77* (9), 2053-2057. doi: 10.1099/0022-1317-77-9-2053

Sánchez-Matamoros, A., Sánchez-Vizcaíno, J., Rodríguez-Prieto, V., Iglesias, E., & Martínez-López, B. (2016). Identification of suitable areas for African horse sickness virus infections in Spanish equine populations. *Transbound Emerg Dis*, *63* (5), 564–573. doi: 10.1111/tbed.12302

Scanlen, M., Paweska, J., Verschoor, J., & Van Dijk, A. (2002). The protective efficacy of a recombinant VP2-based African horse sickness subunit vaccine candidate is determined by adjuvant. *Vaccine*, *20* (7-8), 1079-1088. doi: 10.1016/s0264-410x(01)00445-5

Stone-Marschat, M., Moss, S., Burrage, T., Barber, M., Roy, P., & Laegreid, W. (1996). Immunization with VP2 is sufficient for protection against lethal challenge with African horse sickness virus Type 4. *Virology* , *220* (1), 219-222. doi: 10.1006/viro.1996.0304

van Rijn, P. A., Maris-Veldhuis, M. A., Potgieter, C. A., & Van Gennip, R. G. (2018). African horse sickness virus (AHSV) with a deletion of 77 amino acids in NS3/NS3a protein is not virulent and a safe promising AHS Disabled Infectious Single Animal (DISA) vaccine platform. *Vaccine*, *36* (15), 1925-1933. doi: 10.1016/j.vaccine.2018.03.003

Van Der Saag, M., Ward, M., & Kirkland, P. (2017). Application of an embryonated chicken egg model to assess the vector competence of Australian Culicoides midges for bluetongue viruses. *Med Vet Entomol* , *31* (3), 263–271. doi: 10.1111/mve.12231

Venter, G., Graham, S., & Hamblin, C. (2000). African horse sickness epidemiology: vector competence of South African Culicoides species for virus serotypes 3, 5 and 8. *Med Vet Entomol* , *14* (3), 245–250. doi: 10.1046/j.1365-2915.2000.00245.x

Venter, G., Groenewald, D., Paweska, J., Venter, E., & Howell, P. (1999). Vector competence of selected south african culicoides species for the bryanston serotype of equine encephalosis virus. *Med Vet Entomol* , *13* (4), 393–400. doi: 10.1046/j.1365-2915.1999.00188.x

Venter, G., & Paweska, J. (2007). Virus recovery rates for wild-type and live-attenuated vaccine strains of African horse sickness virus serotype 7 in orally infected South African Culicoides species. *Med Vet Entomol* , *21* (4), 377–383. doi: 10.1111/j.1365-2915.2007

.00706.x

Venter, G., Wright, I., & Paweska, J. (2010). A comparison of the susceptibility of the biting midge *Culicoides* *imicola* to infection with recent and historical isolates of African horse sickness virus. *Med Vet Entomol* , *24* (3), 324–328. doi: 10.1111/j.1365-2915.2010.00895.x

Venter, G., Wright, I., Van Der Linde, T., & Paweska, J. (2009). The oral susceptibility of South African field populations of Culicoides to African horse sickness virus. *Med Vet Entomol* , *23* (4), 367–378. doi: 10.1111/j.1365-2915.2009.00829.x

von Teichman, B. F., Dungu, B., & Smit, T. K. (2010). In vivo cross-protection to African horse sickness Serotypes 5 and 9 after vaccination with Serotypes 8 and 6. *Vaccine*, *28* (39),

6505-6517. doi: 10.1016/j.vaccine.2010.06.105

Whitworth, S. (1930). Memorandum on horse-sickness immunization. In Papers veterinary section, no. 30. Union of South Africa, Dept. of Agriculture; Pretoria: University of Pretoria, Dept. of Library Services (Digital publisher). Retrieved from [https://repository.up.ac.za/bitstream/handle/2263/](https://repository.up.ac.za/bitstream/handle/2263/13834/30_paper30_whitworth.pdf?sequence=1) [13834/30_paper30_whitworth.pdf?sequence=1](https://repository.up.ac.za/bitstream/handle/2263/13834/30_paper30_whitworth.pdf?sequence=1) (Accessed: 2021-09-28)

Wittmann, E., Mellor, P., & Baylis, M. (2002). Effect of temperature on the transmission of orbiviruses by the biting midge, *Culicoides sonorensis*. *Med Vet Entomol* , *16* (2), 147-156. doi: 10.1046/j.1365-2915.2002.00357.x
